# Influenza Vaccine Immunogenicity in Hemodialysis Patients

**DOI:** 10.3390/vaccines14010063

**Published:** 2026-01-04

**Authors:** Anna-Polina Shurygina, Ekaterina Romanovskaya-Romanko, Vera Krivitskaya, Mariia Sergeeva, Janna Buzitskaya, Kirill Vasilyev, Marina Shuklina, Konstantin Vishnevskii, Dmitry Smotrov, Aleksey Tutin, Dmitry Lioznov, Marina Stukova

**Affiliations:** 1 Smorodintsev Research Institute of Influenza, Ministry of Health of the Russian Federation, 197022 St. Petersburg, Russia; 2 St. Petersburg State Budgetary Healthcare Institution “City Hospital No. 15”, 198205 St. Petersburg, Russia; 3 Dialysis Department, North-Western State Medical University Named After I. I. Mechnikov, 191015 St. Petersburg, Russia; 4 Kupchino Outpatient Dialysis Center, 192102 St. Petersburg, Russia

**Keywords:** influenza inactivated vaccines, end-stage renal disease, hemodialysis, longitudinal humoral and cellular immune response

## Abstract

**Background:** Patients with end-stage renal disease (ESRD) on hemodialysis are at increased risk for severe influenza, and underlying immune dysfunction may limit vaccine-induced protection. **Methods:** This observational open-label study evaluated immune responses in 93 hemodialysis patients vaccinated with seasonal inactivated influenza vaccine (IIV) during the 2019–2020 (n = 22) and 2023–2024 (n = 71) seasons. Immune responses were comprehensively assessed using hemagglutination inhibition and microneutralization assays to measure antibody levels, together with flow cytometry analysis of key immune cell populations, including plasmablasts, T-follicular helper cells (Tfh), and effector memory T cells (Tem). **Results:** During the 2019–2020 season, antibody responses in hemodialysis patients were comparable to those in healthy volunteers in both younger (18–60 years) and older (over 60) age groups. By day 7 post-vaccination, there was a pronounced increase in activated Tfh1 cells, coinciding with a surge in plasmablasts and a rise in antigen-specific B cells. This was accompanied by a T-cell response mediated by IFNγ-producing and polyfunctional CD4+ Tem cells. In the 2023–2024 season, revaccination was associated with higher baseline antibody levels but did not alter subsequent response kinetics to A/H1N1pdm, A/H3N2, and B/Yamagata antigens. In contrast, responses to B/Victoria were higher in revaccinated patients throughout the entire observation period. **Conclusions:** Our findings confirm that standard-dose IIV vaccination is beneficial for hemodialysis patients, inducing robust and adequate humoral and T-cell immune responses.

## 1. Introduction

Chronic kidney disease (CKD) is a major public health challenge, affecting more than 10% of the world population, or over 800 million individuals [1]. After cardiovascular complications, infections are the leading cause of hospitalization and mortality in patients with end-stage renal disease (ESRD), especially those receiving hemodialysis (HD) [2]. Mortality from pulmonary infections in patients with CKD is tenfold higher than in the general population [3]. Thus, such patients face a higher risk of severe influenza infection and related complications [4].

The increased susceptibility of patients with ESRD to infections could be caused by multiple factors [5], including immune dysfunction, old age, and comorbid conditions, such as diabetes, mineral and bone disorders, iron metabolism disorders, and anemia. Additional contributors include invasive dialysis procedures, disruption of skin and mucous barriers, and constant exposure to nosocomial flora.

Immune dysfunction in patients with ESRD is characterized by the coexistence of immune activation, manifested as systemic inflammation, and immunodeficiency [6]. Systemic inflammation is the main factor in the development of atherosclerosis, while immunodeficiency compromises host defense against infections and, as a result, determines a more complicated, severe disease course and increased mortality in infectious diseases. Immunodeficiency also underlies the reduced magnitude of post-vaccination immune responses in patients with ESRD [7]. The severity of immunodeficiency increased with advancing uremia [8,9,10], with both innate and adaptive immune responses affected.

End-stage renal failure is accompanied by immunological aging of both CD4+ and CD8+ T cell populations. Lymphopenia, observed in many studies, primarily reflects a decrease in naive T-lymphocytes caused by inhibition of T-cell production in the thymus and increased apoptosis [6,9]. At this stage of CKD, the B-cell population is also depleted, which leads to reduced effectiveness of humoral immune response [11].

Uremia is not the only cause of immunodeficiency in patients with CKD. Hemodialysis is an independent factor that can negatively affect the number of dendritic cells, the number and phagocytic activity of granulocytes, as well as the number and suppressor activity of regulatory T cells [12,13]. Another independent contributor to a weak immune response is patient age, as ESRD occurs predominantly in individuals over 60 years compared with the general population [14].

Although evidence regarding the safety and protective efficacy of influenza vaccines in patients with CKD is limited and of variable quality, annual influenza vaccination is mandatory for all patients with CKD [15,16]. Given the substantial risk posed by influenza in this population, even a moderate level of preventive effectiveness can be considered meaningful [15]. Multiple studies have shown that influenza vaccination in patients with CKD receiving hemodialysis can reduce the risk of pneumonia/influenza and other diseases, lower the likelihood and duration of hospitalization (including intensive care admission), and decrease mortality, especially among older adults [17]. Influenza vaccination in elderly patients with CKD is associated with reduced rates of hospitalizations for heart failure [18], as well as with lower risk of acute coronary syndrome [19], peripheral arterial occlusive disease [20], and dementia [21].

According to international recommendations (KDIGO 2012), seasonal influenza prevention should be carried out annually using inactivated influenza vaccines (IIVs) in all patients with CKD, irrespective of disease stage, dialysis status, or kidney transplantation. In patients over 65 years of age, use of higher-dose IIV formulations, such as Fluzone High-Dose or FluBlok (Sanofi Pasteur, Lyon, France), may be recommended [22]. However, evidence suggests that repeated annual IIV vaccination can blunt vaccine effectiveness [23,24].

In this study, we evaluated post-vaccination humoral and cellular immune responses to IIVs available in Russia during the 2019–2020 epidemic season in 22 hemodialysis patients. Immune responses were monitored for 6 months after vaccination, and immunological efficacy of IIVs was compared with the data for healthy volunteers without CKD in two age groups: 18–60 years (n = 34) and over 60 years (n = 42).

During the 2023–2024 season, the impact of prior vaccination was assessed by measuring antigen-specific antibody responses and induced IFNγ production in whole-blood samples. The cohort of 71 hemodialysis patients was divided into groups based on their vaccination history: individuals not vaccinated with IIV during the two preceding epidemic seasons (n = 28); individuals vaccinated with IIV in the immediately preceding season (2022–2023; n = 34); and individuals vaccinated in both of the two prior seasons (2021–2022 and 2022–2023; n = 9).

## 2. Materials and Methods

### 2.1. Study Design and Populations

Between October 2019 and April 2020, 22 adults undergoing hemodialysis at the St. Petersburg State Budgetary Healthcare Institution “City Hospital No. 15”, Russia, were enrolled in an observational open-label study conducted by the Smorodintsev Research Institute of Influenza, Ministry of Health of the Russian Federation. Participants were 18 years of age or older and had a documented influenza vaccination history. Inclusion criteria were: (i) no influenza vaccination during the preceding season; (ii) receipt of trivalent (TIV) or quadrivalent influenza vaccine (QIV) in the 2019/2020 flu season; (iii) willingness to provide blood samples to monitor humoral and cellular immune response at predefined time points over a 6-month observation period.

Blood samples for serum and PBMC isolation were collected from hemodialysis patients on an inpatient basis. Samples were taken pre-vaccination (day 0) and then at 7 days, 21 (or 28) days, 3 months, and 6 months post-vaccination. Two groups of healthy volunteers, 18–60 years old (n = 34) and over 60 years old (n = 42), were enrolled as outpatients in the clinical department of the Smorodintsev Research Institute of Influenza, Ministry of Health of the Russian Federation. The same inclusion criteria were applied, except that blood was collected only for serum samples on days 0 and 21.

In 2023, we additionally recruited a new cohort of 71 hemodialysis volunteers who were followed at the Kupchino outpatient dialysis center (St. Petersburg, Russia). This cohort was stratified as follows: 28 patients (39.44%) had not received any inactivated influenza vaccine (IIV) in the two preceding flu epidemic seasons; 34 patients (47.89%) had received IIV in the preceding season (2022/2023); and 9 patients (12.67%) had been vaccinated in both of the prior seasons (2021/2022; 2022/2023). The latter were excluded from the comparative analysis due to insufficient sample size.

### 2.2. Immunization

During the 2019/2020 flu season in the Russian Federation, two subunit adjuvanted TIVs were available for immunization: “Grippol plus”(GP; NPO Petrovax, Moscow, Russia), which contained 5 μg of influenza virus hemagglutinin (HA) from each of the A/H1N1, A/H3N2 and B/Victoria strains, together with the adjuvant Polyoxidonium (500 μg per dose), and “Sovigripp”(SG; NPO Microgen, Moscow, Russia), which contained 5 μg of HA from A/H1N1 abd A/H3N2, 11 μg of HA from B/Victoria, and the adjuvant Sovidon (500 μg per dose). In addition, during this season, the quadrivalent non-adjuvanted split vaccine “Ultrix Quadri” (UQ; FORT LLC, Moscow, Russia), containing 15 μg of HA from each of the A/H1N1, A/H3N2, B/Victoria, and B/Yamagata strains per dose, was used for immunization [25,26]. The strain composition of these vaccines corresponded to WHO recommendations and included A/Brisbane/02/2018 (A(H1N1)pdm09), A/Kansas/14/2017 (A(H3N2)), B/Colorado/06/2017 (B/Victoria), and B/Phuket/3073/13 (B/Yamagata; quadrivalent vaccine only).

In 2023, all 71 hemodialysis patients received the quadrivalent inactivated vaccine (QIV) Flu-M Tetra. The strain composition corresponded to WHO recommendations and included A/Victoria/4879/2022 (A(H1N1)pdm09), A/Thailand/8/2022 (H3N2), B/Austria/1359417/2021 (B/Victoria), and B/Phuket/3073/13 (B/Yamagata). This composition differed from that of the 2022–2023 season vaccine with respect to the influenza A virus components (A/Victoria/2570/2019 (A(H1N1)pdm09), A/Darvin/09/2021(A(H3N2)).

Vaccination was performed after informed consent was obtained and was carried out by trained medical personnel. Each vaccine was administered into the deltoid muscle (upper third of the outer arm) after the completion of the hemodialysis procedure. Prior to vaccine administration, participants underwent thermometry, blood pressure measurement, an interview, and a clinical examination conducted by the physician responsible for vaccination. After immunization, participants were observed for 30 min by a physician and subsequently for at least 5 days, depending on the timing of post-vaccination reaction recording, under outpatient medical supervision.

### 2.3. Antibody Response Assessment

Systemic antibody responses to vaccination were assessed in serum samples using a hemagglutination inhibition (HAI) assay and a microneutralization assay (MNA). The HAI assay was performed in accordance with established national guidelines [27], whereas the MNA was conducted following standard WHO protocols [28]. For the 2019/2020 season, the assays used the following diagnostic strains as antigens: A/Brisbane/02/2018 (A(H1N1)pdm09), A/Kansas/14/2017 (A(H3N2)), B/Colorado/06/2017 (B/Victoria lineage), and B/Phuket/3073/13 (B/Yamagata lineage). For the 2023/2024 season, the antigen panel was updated to include A/Victoria/4879/2022 (A(H1N1)pdm09), A/Thailand/8/2022 (A(H3N2)), B/Austria/1359417/2021 (B/Victoria lineage), and B/Phuket/3073/13 (B/Yamagata lineage).

Antibody titers measured by HAI and MNA were defined as the reciprocal of the highest serum dilution that inhibited hemagglutination or cytopathic effect (CPE), respectively. Samples that showed no inhibition at the initial dilution of 1:10 were assigned a titer of 5. The fold increase in antibody titer was calculated as the ratio of the post-vaccination titer (Day X) to the baseline titer (Day 1). Seroconversion was defined as a ≥4-fold rise in antibody titer, and individuals meeting this criterion were classified as responders. The responder rate for each group was calculated based on seroconversion detected on any post-vaccination day assessed.

### 2.4. Peripheral Blood Mononuclear Cell (PBMCs) Isolation and Cryopreservation

For PBMCs isolation, venous blood samples were collected in sodium heparin sulfate tubes. PBMCs were isolated by sedimentation in a ficoll density gradient within 4 h of blood collection [29] and then frozen and stored in liquid nitrogen until use.

### 2.5. Intracellular Cytokine Staining (ICS)

To assess influenza-specific T-cell responses, thawed PBMCs were stimulated with influenza split vaccine monocomponents corresponding to A/H1N1pdm09, A/H3N2, B/Victoria, and B/Yamagata (5 µg/well), kindly provided by SPBNIIVS. The frequency of antigen-specific cytokine-producing Tem lymphocytes was evaluated using an intracellular cytokine staining (ICS) protocol with the BD Fixation/Permeabilization kit (BD, San Diego, CA, USA) according to the manufacturer’s instructions. Panels of fluorescently labeled antibodies targeting the surface markers CD3, CD4, CD8, CD45RA, and CCR7 (Biolegend, San Diego, CA, USA) and the intracellular cytokines IFN-γ, IL-2, and TNF-α (Biolegend, USA) were used. The gating strategy is shown in the Supplementary (Figure S1). Data were acquired using a Cytoflex flow cytometer (Beckman Coulter, Brea, CA, USA). Flow cytometry data were processed using CytExpert software (Available online: https://www.mybeckman.nl/flow-cytometry/research-flow-cytometers/cytoflex/software, (accessed on 29 December 2025)) (Beckman Coulter, USA) and Kaluza 2.0 (Beckman Coulter, Brea, CA, USA).

### 2.6. T-Follicular Helper Cells (Tfh) Evaluation

Peripheral Tfh subpopulations in cryopreserved PBMCs were phenotyped using the following markers: CD3, CD4, CXCR5, ICOS, CCR6, CXCR3, CD27, CD45RA, CCR7, CD62L, and PD1 (Figure S2).

### 2.7. B-Cell Evaluation

B-cell responses were characterized by flow cytometry in cryopreserved PBMCs. The following markers were used for immunophenotyping: CD3, CD20, CD27, CD38, IgD, and IgA. Within the CD3^−^CD19^+^ B cell gate, the following subsets were identified: naïve B cells (CD20*^+^*CD27*^−^*IgD*^+^*), non-switched memory B cells (CD20*^+^*CD27*^+^*IgD*^+^*), switched memory B cells (CD20*^+^*CD27*^+^*IgD*^−^*), effector memory B cells (CD20*^+^*CD27*^−^*IgD*^−^*), and plasmablasts (CD20*^−^*CD38^hi^CD27^hi^) (Figure S3).

To assess B-cell activation, we quantified the proportion of CD38+ B cells within the naïve, effector memory, and switched and non-switched memory B cell subsets. (Figure S2). Antigen-specific B-cell responses were evaluated using an ELISPOT assay with commercial kits (Mabtech, Nacka Strand, Sweden), according to a protocol adapted from Haralambieva et al. [30]. Briefly, cryopreserved PBMCs were thawed and cultured for 72 h in the presence of recombinant human IL-2 (10 ng/mL) and the TLR7/8 agonist R848 (1 µg/mL). Cells were then transferred to membrane plates (Millipore, Billerica, MA, USA) pre-coated with either viral antigens or a capture antibody.

Coating antigens included monocomponents of the influenza split vaccine (A/H1N1pdm09, A/H3N2, B/Victoria, and B/Yamagata) at a concentration of 5 µg per well. An anti-human IgG capture antibody (clone MT91/45, 15 µg/mL) served as a positive control for total IgG-secreting cells, while wells containing DPBS alone were used as a negative control. Cells were seeded at densities of 2 × 10^5^ cells/well for antigen-specific responses and 1 × 10^4^ cells/well for total IgG quantification. After a 20 h incubation at 37 °C with 5% CO_2_, the cells were removed, and the plates were developed according to the manufacturer’s instructions. Antibody-secreting cells (ASCs) were visualized as spots and quantified using an ImmunoSpot S6 Ultimate UV Image Analyzer (CTL, Floral Park, NY, USA). Results are expressed as spot-forming units (SFU) per 2.0 × 10^5^ PBMCs.

### 2.8. Antigen-Specific IFNγ Release in Whole-Blood Cultures

Heparinized whole blood was aliquoted at 50 µL/well into pre-prepared 96-well culture plates containing complete culture medium supplemented with influenza virus antigens A/H1N1pdm09, A/H3N2, B/Victoria, and B/Yamagata (5 µg/well) 150 µL/well. For each sample, wells corresponding to positive controls (non-specific stimulation with PMA + Ionomycin) and negative controls (unstimulated cells in complete culture medium) were included. The samples were incubated at 37 °C, 5% CO_2_ for 24 h, and then supernatants were collected and stored at −70 °C. The level of IFN-γ induction in stimulated samples was determined using a commercial ELISA kit, “Gamma-Interferon-ELISA-BEST” (Vector-BEST, Koltsovo, Russia), according to the manufacturer’s instructions. Optical Density (OD) was measured using a Multiscan Sky High spectrophotometer (Thermo Scientific, Waltham, MA, USA) at a primary wavelength of 450 nm and a reference wavelength of 650 nm.

### 2.9. Statistics

Statistical analysis and data visualization were performed using GraphPad Prism (version 10.1.0) and RStudio Desktop (version 3.3.0). Data are presented as the arithmetic mean ± standard deviation (SD) or standard error of the mean (SEM), or box-and-whiskers plots. For linked samples, the Friedman test followed by a post hoc Dunn’s multiple comparisons test, or RM ANOVA followed by a post hoc Tukey’s multiple comparisons test, was used. Unrelated samples were analyzed using the Kruskal–Wallis test or the unpaired *t*-test. Correlations were assessed using Pearson’s test. Statistical significance was defined as a two-tailed *p*-value < 0.05.

## 3. Results

### 3.1. Study Population

During the 2019/2020 flu season, 22 hemodialysis patients were included in the study (Cohort 2019/2020). Table 1 shows detailed characteristics of the cohort, with the mean age of 64 years and 73% of the participants being over 60 years old. All patients received dialysis therapy three times a week for 4–4.5 h. Hemodialysis efficiency, calculated using the spKt/V single-pool urea distribution model to assess effective urea clearance per dialysis session as a fraction of the urea distribution volume in each patient [31], was satisfactory in all patients (>1.4).

### 3.2. Immunological Efficacy of IIVs in Hemodialysis Patients

**Figure 1:**
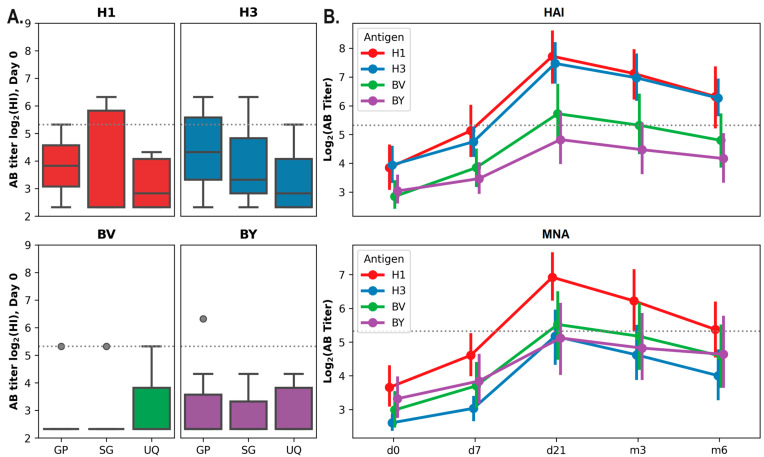
(**A**) Distribution of volunteers (hemodialysis patients, cohort 2019/2020) according to individual antibody titers measured by the HAI assay on Day 0 (before vaccination). Data are presented as box plots, showing the median, interquartile range, and minimum and maximum titer values. The horizontal dotted line indicated a titer of 40, corresponding to the minimum protective AB level. (**B**) Dynamics of virus-specific antibody titers in the sera of vaccinated hemodialysis patients over a 6-month period after vaccination. Graphs show the mean ± SD of log_2_-transformed AB titers measured by HAI and MNA. GP—Grippol Plus, SG—Sovigripp, UQ—Ultrix Quadri. The dots indicate the outliers.

Due to prior influenza infection or vaccination, preexisting antibody (AB) levels against various influenza strains and subtypes can vary markedly between individuals. Among vaccinated hemodialysis patients, both seronegative (antibody titer ≤ 1:20) and seropositive individuals, defined as having a protective baseline titer ≥ 1:40 by HAI, were identified. Before the start of vaccination in autumn 2019, the proportion of seronegative individuals in this cohort was 77% for influenza A/H1N1pdm09 virus, 68% for influenza A/H3N2, 86.4% for B/Victoria, and 95.5% for B/Yamagata virus. There were no statistically significant differences in baseline antibody (AB) titers among volunteers immunized with different vaccines for any of the vaccine components (A/H1N1pdm09, A/H3N2, B/Victoria, B/Yamagata; *p* < 0.05, Kruskal–Wallis test, Figure 1).

The immunological efficacy of all vaccines administered to hemodialysis patients met the criteria established by the European Committee on Patented Medicines (CPMP/BWP/214/1996) for IIVs, using the criteria defined for individuals older than 60 years (Table 2).

Table 3 summarizes neutralizing AB responses following vaccination with the studied IIVs. Notably, neutralizing AB titers were comparable to HA-binding antibody titers only for the A/H1N1pdm09 component, whereas neutralizing antibody titers against A/H3N2 and both influenza B virus components were significantly lower than the corresponding HA-binding antibody titers (Table 3). This pattern was observed not only in hemodialysis patients but also in other volunteer cohorts vaccinated in the 2019–2020 epidemic season in a cross-sectional study [32].

Comparison of immunological efficacy parameters between hemodialysis patients and healthy volunteers without CKD in two age groups revealed that hemodialysis patients responded better to the B/Victoria component (Table 4).

### 3.3. Post-Vaccination Immune Response Dynamics in Hemodialysis Patients

The dynamics of the antibody response in the hemodialysis cohort was evaluated over a 6-month period following vaccination (Figure 1B). Peak titers of hemagglutination-inhibiting (HAI) and neutralizing antibodies (NAbs) were observed at 21 days post-vaccination, followed by a gradual decline. However, for the H1N1pdm09 and H3N2 viruses, HAI antibody titers remained above 40, i.e., above the minimum protective level, six months after vaccination.

**Figure 2:**
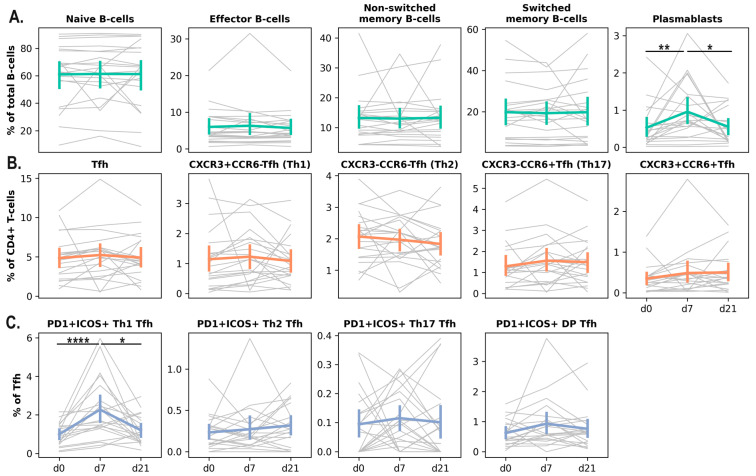
Changes in peripheral blood B and Tfh cell subpopulations at early time points after IIV administration in hemodialysis patients (Cohort 2019/2020; n = 22): B-cell subpopulation dynamics (**A**), Tfh subpopulation dynamics (**B**), and activated Tfh subpopulation dynamics (**C**). Gray lines represent individual patient trajectories; colored lines indicate the mean ± SE. Friedman test followed by a post hoc Dunn’s multiple comparisons test (*: *p* < 0.05; **: *p* < 0.01; ****: *p* < 0.0001).

In addition to assessing the humoral response to IIVs in the hemodialysis cohort, we performed a comprehensive analysis of the cellular immune response. We evaluated the frequencies of plasmablasts (PB), T-follicular helper cells (Tfh), antigen-specific memory B cells, and effector memory CD4^+^ and CD8^+^ T cells (Tem). Vaccination resulted in a significant increase in the proportion of activated (PD1 + ICOS+) Tfh1 cells and plasmablasts in peripheral blood on day 7 after vaccine administration. By day 21, the frequencies of these cell populations returned to baseline levels (Figure 2).

**Figure 3:**
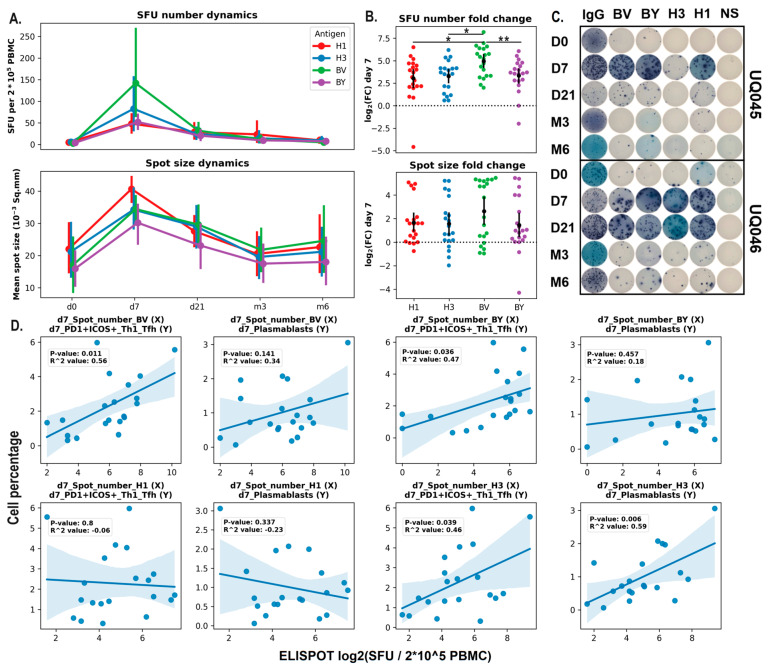
Antigen-specific B cell responses in hemodialysis patients (Cohort 2019/2020; n = 22; ELISPOT). (**A**) Dynamics of the number of antigen-specific antibody-secreting B cells (mean ± SEM) and mean spot size (mean ± SEM). (**B**) Log2 fold change (FC) in the number of antigen-specific antibody-secreting B cells on day 7 relative to pre-vaccination counts (day 0). Data were considered statistically significant at *p* < 0.05, as determined by RM one-way ANOVA followed by Tukey’s multiple comparison test (*: *p* < 0.05; **: *p* < 0.01). (**C**) Representative ELISPOT images from two volunteers. (**D**) Pearson correlation analysis between the percentage of circulating Tfh1 and plasmablasts and the number of antigen-specific B-cells measured by ELISPOT.

The dynamics of antigen-specific B cells in the peripheral blood of hemodialysis patients after vaccination was measured using the ELISPOT assay (Figure 3A–D). Both the number of spots (SFU) and the mean spot size increased significantly on day 7 after vaccination and returned to baseline levels afterwards (d21–m6). The most prominent increase in total SFUs, relative to day 0, was induced by the B/Victoria vaccine component compared to the H1 and H3 components (Figure 3A,B).

Pearson correlation analysis was performed to assess associations between changes in immune cell populations in peripheral blood and antigen-specific responses measured by ELISPOT (Figure 3D). Statistically significant correlations were observed for parameters that increased on day 7 after vaccine administration. The frequencies of activated (PD1 + ICOS+) Tfh1 and plasmablasts were positively correlated with the mean SFU numbers for the H3 and B/Victoria vaccine components. In contrast, no significant correlations were identified for the H1 and B/Yamagata antigens.

Next, we analyzed the magnitude, functional characteristics, and duration of post-vaccination antigen-specific (A/H1N1pdm09, A/H3N2, B/Victoria, and B/Yamagata) T-cell responses to IIVs in hemodialysis patients by flow cytometry on cryopreserved PBMC samples collected before vaccination (Day 0) and at 7 days, 21 days, and 6 months after vaccination, regardless of the vaccine type.

**Figure 4:**
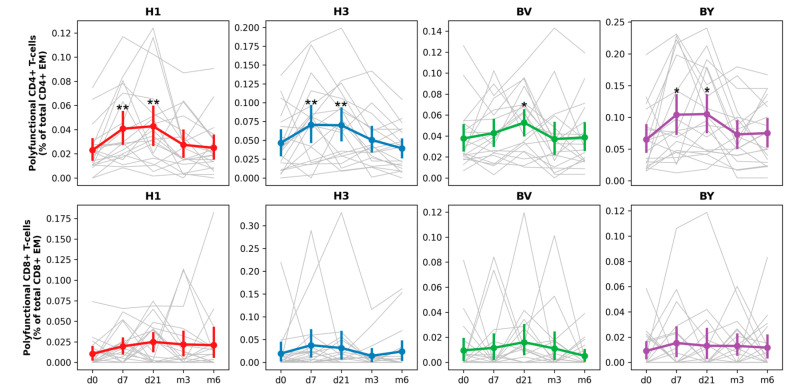
Dynamics of post-vaccination CD4+ and CD8+ T-cell responses in hemodialysis patients (Cohort 2019/2020; n = 22). Cumulative frequencies of antigen-specific polyfunctional (IFNγ + IL2 + TNFα-, IFNγ + IL2-TNFα+, IFNγ-IL2 + TNFα+, IFNγ + IL2 + TNFα+) cytokine-producing CD4+ and CD8+ Tem cells are expressed as % of total Tem (CD45RA-CCR7-) cells. Asterisks (*) indicate statistically significant differences relative to day 0, as determined by the Friedman test followed by a post hoc Dunn’s multiple comparisons test (*: *p* < 0.05; **: *p* < 0.01). Gray lines represent individual patient trajectories; colored lines show the mean ± SE.

Vaccination induced an increase in the total number of antigen-specific polyfunctional CD4+ Tem cells at 7 and 21 days after administration (Figure 4). We observed the strongest immune responses when cells were stimulated with influenza A/H1N1pdm09, A/H3N2, and B/Yamagata antigens. CD8+ Tem responses were more modest.

Thus, IIV vaccination induced a T-cell immune response in hemodialysis patients, which was predominantly characterized by the formation of polyfunctional CD4+ effector T-lymphocytes.

### 3.4. Effect of Repeated IIV Administration on the Post-Vaccination Immune Response

Originally, the study was intended to follow the same cohort of hemodialysis patients through several consecutive influenza epidemic seasons but was interrupted during the COVID-19 pandemic in 2020. At that time, the dialysis center participating in the study was converted into an infectious disease hospital, which led to the redistribution of the patient cohort to other centers. In 2023, we recruited a new cohort of 71 hemodialysis volunteers, categorized as follows: the 1Y group included 28 patients (39.44%) who had not received any IIVs during the previous two influenza epidemic seasons; 2Y—34 patients (47.89%) who had received IIV in the previous season (2022/2023); and 3Y—9 patients (12.67%) who had been vaccinated in each of the previous two seasons (2021/2022 and 2022/2023). Patients in the 3Y group were excluded from the comparative analysis because the sample size was insufficient.

Vaccine strain composition corresponded to WHO recommendations for 2022 and included A/Victoria/2570/2019 (A(H1N1)pdm09), A/Darvin/09/2021(A(H3N2)), B/Austria/1359417/2021 (B/Victoria), and B/Phuket/3073/13 (B/Yamagata). In 2023, the A(H1N1)pdm09 component was replaced with A/Victoria/4879/2022, and the H3N2 component with A/Thailand/8/2022, while both influenza B virus strains remained the same. All 71 hemodialysis patients received the quadrivalent IIV Flu-M Tetra. Detailed characteristics of the cohort are shown in Table 5.

Vaccine immunogenicity, assessed by HAI and MNA, met CPMP criteria for all vaccine components, regardless of vaccination status in the preceding season (Table 6 and Table 7). According to the HAI assay using antigens specified for 2023, baseline seroprotection rates were significantly higher in the repeat-vaccine group (2Y) than in the 1Y group only for the B/Victoria component. In contrast, MNA showed higher baseline neutralizing antibody titers against all vaccine components in the repeat-vaccine group.

**Figure 5:**
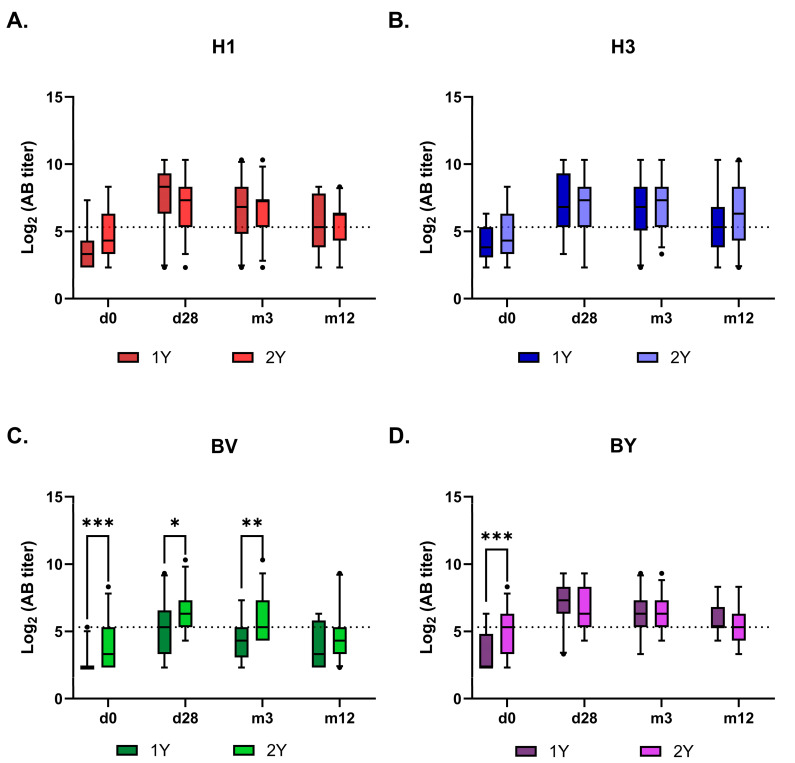
Kinetics of virus-specific antibody titers in sera from vaccinated hemodialysis patients over 12 months after vaccination. Antibody levels measured by HIA are presented as box-and-whiskers plots representing the 5–95% percentiles. Panels show responses to (**A**) H1—A/Victoria/4879/2022 (A(H1N1)pdm09); (**B**) H3—A/Thailand/8/2022 (H3N2); (**C**) BV—B/Austria/1359417/2021 (B/Victoria); (**D**) BY—B/Phuket/3073/2013 (B/Yamagata). 1Y indicates the group of hemodialysis patients vaccinated in the 2023/2024 season, and 2Y indicates the group of hemodialysis patients vaccinated in both the 2022/2023 and 2023/2024 seasons. Statistical significance was defined as *p* < 0.05 and evaluated using RM two-way ANOVA followed by Tukey’s multiple comparison test (*: *p* < 0.05, **: *p* < 0.01, ***: *p* < 0.001). The dots indicate the outliers.

**Figure 6:**
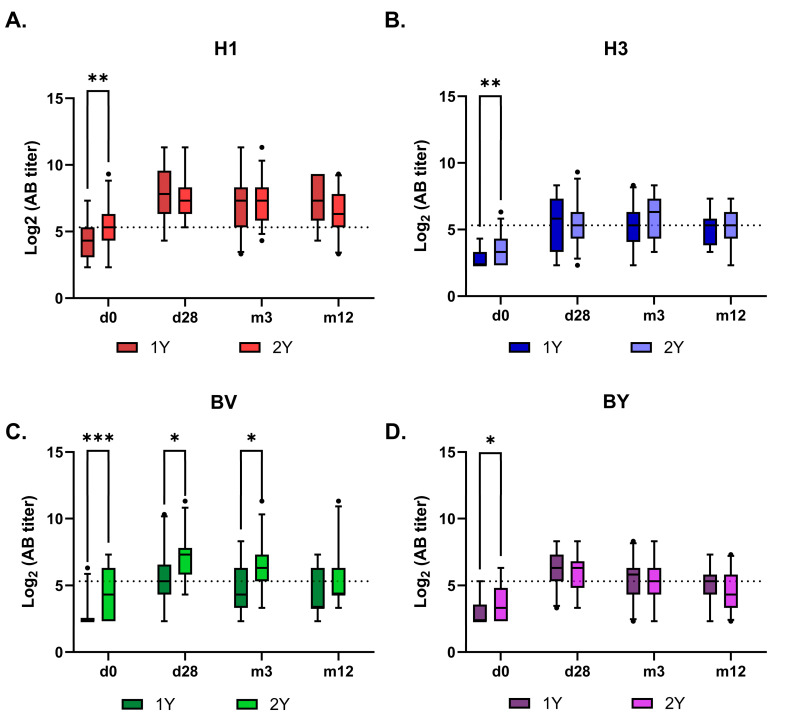
Kinetics of virus-specific neutralizing antibody titers in sera from vaccinated hemodialysis patients over 12 months after vaccination. Antibody levels measured by MNA are presented as box-and-whiskers plots representing the 5–95% percentiles. Panels show responses to (**A**) H1—A/Victoria/4879/2022 (A(H1N1)pdm09); (**B**) H3—A/Thailand/8/2022 (H3N2); (**C**) BV—B/Austria/1359417/2021 (B/Victoria); (**D**) BY—B/Phuket/3073/2013 (B/Yamagata). 1Y indicates the group of hemodialysis patients vaccinated in the 2023/2024 season, and 2Y indicates the group of hemodialysis patients vaccinated in both the 2022/2023 and 2023/2024 seasons. Statistical significance was defined as *p* < 0.05 and evaluated using RM two-way ANOVA followed by Tukey’s multiple comparison test (*: *p* < 0.05, **: *p* < 0.01, ***: *p* < 0.001). The dots indicate the outliers.

Antibody kinetics after vaccination were similar between the 1Y and 2Y groups across all vaccine antigens, except for the B/Victoria component (Figure 5 and Figure 6). The response to this component remained higher in the 2Y group for at least three months post-vaccination, converging with the 1Y level only after one year. Interestingly, no such booster effect of repeated vaccination was observed for the B/Yamagata component.

The impact of vaccination on the T-cell immune response was assessed by measuring IFN-γ production in antigen-stimulated whole-blood samples. This approach has previously been described as a simple and informative method for evaluating T-cell responses to influenza vaccination [33].

**Figure 7:**
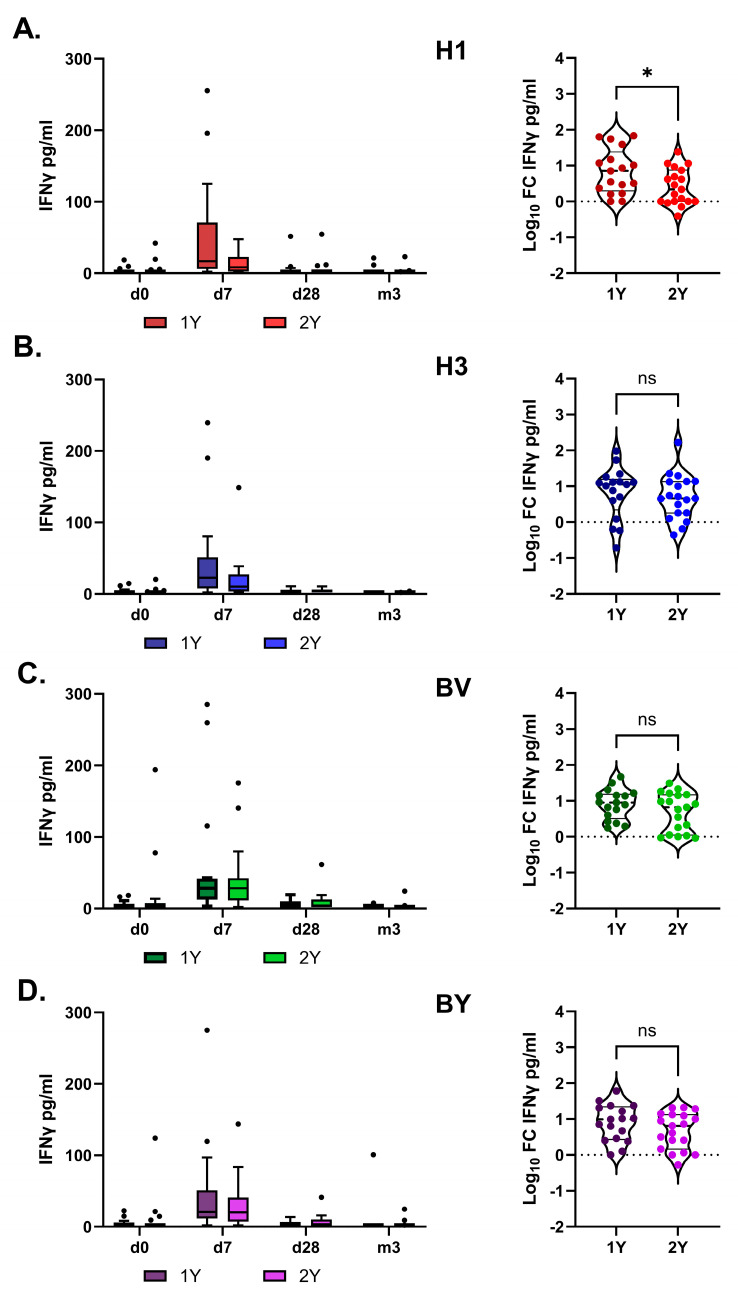
IFNγ production in whole-blood cell cultures stimulated with split influenza vaccine antigens. Panels show responses to (**A**) H1—A/Victoria/4879/2022 (A(H1N1)pdm09); (**B**) H3—A/Thailand/8/2022 (H3N2); (**C**) BV—B/Austria/1359417/2021 (B/Victoria); (**D**) BY—B/Phuket/3073/2013 (B/Yamagata). 1Y indicates the group of hemodialysis patients vaccinated in the 2023/2024 season, and 2Y indicates the group of hemodialysis patients vaccinated in both the 2022/2023 and 2023/2024 seasons. The left column shows antigen-specific IFNγ concentrations (pg/mL) after subtraction of baseline values. The right column shows the post-vaccination log_10_ fold change (FC) in IFNγ concentrations on day 7 relative to day 0. Statistical significance was defined as *p* < 0.05 and evaluated using an unpaired *t*-test (*: *p* < 0.05). The dots indicate the outliers.

IFN-γ production peaked on day 7 after vaccination and did not differ significantly between the study groups overall (RM two-way ANOVA followed by Tukey’s multiple comparison test). However, in the 1Y group, the fold increase in IFN-γ levels in response to H1 antigen stimulation was higher than in the 2Y group (Figure 7).

## 4. Discussion

Seasonal influenza contributes significantly to morbidity and mortality among immunocompromised individuals, including patients with CKD [4]. Despite conflicting data on the effectiveness of influenza vaccination in patients with CKD, particularly those receiving hemodialysis, its clinical importance appears unequivocal [15,34]. In patients with eGFR ≥ 30 mL/min/1.73 m^2^, influenza vaccination is associated with lower hospitalization rates and a reduced risk of pneumonia [35]. Furthermore, several small cohort studies suggest that annual influenza vaccination is associated with reduced risks of heart failure-related hospitalization [18], acute coronary syndrome [19], and dementia [21] in elderly patients with CKD.

The Practical Guide to vaccination in all stages of CKD, including patients treated by dialysis or kidney transplantation, states that seasonal inactivated influenza vaccine should be administered annually to all adult patients with CKD, whether or not these patients are undergoing dialysis or have received a kidney transplant [22].

Several strategies have been proposed to improve IIV effectiveness in hemodialysis patients, including high-dose vaccines, booster doses, and adjuvanted IIVs. High-dose IIV has been shown to enhance immunogenicity without increasing serious adverse events [36] and, according to McGrath et al., is associated with lower hospitalization rates compared to standard-dose vaccination in hemodialysis patients, particularly in individuals older than 65 years [37]. However, in a subsequent study, high-dose influenza vaccination has not demonstrated significant advantages over standard-dose vaccination in preventing mortality or hospitalizations in this population, raising concerns regarding cost-effectiveness given its higher cost and potential side effects [38].

Booster vaccination with IIV is a longstanding recommendation for enhancing protection in patients with chronic renal disease. Despite this, the clinical efficacy of a booster strategy has been debated, with a subsequent meta-analysis failing to provide conclusive supporting evidence [39].

The recent COVID-19 pandemic has yielded important insights into vaccine effectiveness in the hemodialysis population. As with influenza, hemodialysis patients infected with SARS-CoV-2 are at increased risk of severe disease and mortality, partly due to their frequently immunocompromised status [40]. However, evidence regarding COVID-19 vaccine effectiveness in this group remains inconsistent, with several studies reporting a potentially diminished response compared to the general population [41,42,43,44]. Factors associated with weaker humoral responses in these patients include advanced age, hypoalbuminemia, lymphopenia, intravenous administration of iron at higher doses, and elevated body mass index (BMI) [45]. These observations imply that vaccination effectiveness in hemodialysis patients is likely multifactorial and may involve aspects of the dialysis procedure itself. Supporting this, Tadashi Tomo et al. [46] reported comparatively lower responses to influenza vaccination in hemodialysis patients compared with healthy controls. Notably, this study demonstrated that modulation of a technical treatment parameter—specifically, replacement of polysulfone (PS) dialyzer membranes with polymethylmethacrylate (PMMA)—influences the magnitude of the vaccination response.

In this observational open-label study, we show that standard-dose vaccination induces immune responses in hemodialysis patients that meet the European Committee for Proprietary Medicinal Products (CPMP/BWP/214/1996) criteria for the relevant age category across all studied IIV vaccine components. In addition to a humoral immune response, vaccination triggered a coordinated cellular immune response characterized by a pronounced increase in activated Tfh1 cells, a corresponding surge in plasmablasts, and a rise in antigen-specific B cells on day 7 post-vaccination. This was followed by a T-cell response mediated predominantly by CD4+ effector memory T cells.

We also show that both the magnitude and duration of the humoral immune response in hemodialysis patients were not inferior to those observed in a general population of volunteers initially vaccinated during the 2019–2020 epidemic season [32].

Our findings are consistent with the results published by Johan Scharpé et al. [47] and later by Christos Pleros et al. [48]. Both studies report that influenza vaccination is as effective in hemodialysis patients as in healthy volunteers. Except for serum ferritin levels, none of the investigated parameters of nutrition, inflammation, or dialysis adequacy and modality had a significant impact on immune responses [47]. We suggest that modern, effective dialysis therapy contributes not only to the correction of renal insufficiency but also to partial compensation of CKD-associated immunodeficiency.

On the other hand, Jaromír Eiselt and coauthors reported lower influenza vaccine-induced antibody production in hemodialysis patients than in controls, indicating that previous vaccination history and age are stronger predictors of immune response to influenza vaccine than inflammation and iron status in this population [49].

In recent years, researchers have actively debated the influence of pre-existing antiviral immunity on vaccine-induced responses, particularly in the context of repeated vaccination. Numerous studies indicate that consecutive annual influenza vaccination can reduce vaccine effectiveness [23,24]. One possible explanation for this phenomenon was offered by proponents of the “antigenic distance” hypothesis in the 1990s [50]. They demonstrated that when viral components used in sequential vaccinations are antigenically similar, re-vaccination preferentially reactivates memory B cells generated during the initial exposure, limiting the induction of de novo B-cell responses against newly introduced antigens. This process, termed “negative interference”, reduces the apparent efficacy of repeated vaccination while still providing protection against antigenically similar strains through pre-existing antibodies. By contrast, if the antigenic distance between sequential vaccine strains is sufficiently large, the immune system efficiently recognizes the new antigen upon re-vaccination. This leads to robust induction of strain-specific protective antibodies and memory B cells. To date, relatively few studies have validated this concept of reduced efficacy with repeated vaccination [51,52].

In our study, we observed no evidence of negative interference associated with repeated immunization in hemodialysis patients. Repeated vaccination did not impair antibody kinetics or antigen-specific IFNγ production. On the contrary, the re-vaccinated group showed significantly higher neutralizing antibody activity against all vaccine antigens. We attribute this effect to the minimal antigenic distance between the vaccine strains used in the 2022/2023 and 2023/2024 seasons. The magnitude and dynamics of the antibody response to influenza A viruses and influenza B/Yamagata were comparable between individuals with and without prior-season vaccination. The antibody responses to the B/Victoria component remained significantly higher in the group vaccinated in two consecutive seasons for at least three months post-vaccination. Both groups mounted robust T-cell response (IFN-γ production), with only a modestly higher fold-increase in IFN-γ production to H1 in first-time vaccinees.

In summary, although immunocompromised individuals such as hemodialysis patients carry a significant influenza burden, the body of evidence supports annual influenza vaccination as a crucial and effective protective strategy. Consistent with recent research, our study demonstrates that hemodialysis patients can mount robust and adequate humoral and T-cell immune responses to standard-dose IIVs. These findings suggest that modern dialysis therapy may effectively mitigate the immunodeficiency associated with chronic kidney disease. We contend that the replication of a general immune response pattern in hemodialysis patients across distinct epidemic seasons strengthens the validity of our findings.

## Figures and Tables

**Table 1:** Baseline characteristics of study subjects (hemodialysis patients, cohort 2019/2020).

Demographic Characteristics	Total, n = 22
Age, mean (min–max)	64 (27–83)
Female, n (%)	5 (22.73)
Male, n (%)	17 (77.27)
**Primary kidney disease**	
Polycystic kidney disease, n (%)	8 (36.36)
Abnormality of kidney development, n (%)	4 (18.18)
Chronic glomerulonephritis, n (%)	4 (18.18)
Chronic tubulointerstitial nephritis, n (%)	3 (13.64)
Type 1 diabetes, n (%)	1 (4.55)
Gouty nephritis, n (%)	1 (4.55)
Nephrectomy, n (%)	1 (4.55)
Time on dialysis, years, mean (min-max)	6.1 (0.2–18.2)
spKt/V, mean (95%CI)	1.47 (1.37–1.58)
**Laboratory parameters**	
Hemoglobin, g/L, mean (95%CI)	113.9 (107.1–120.6)
Albumine, g/L, mean (95%CI)	37.8 (36.82–38.78)
P, mmol/L, mean (95%CI)	2.67 (2.49–2.85)
Ca, mmol/L (95%CI)	2.16 (2.05–2.27)
**Vaccination**	
Sovigripp, n (%)	7 (31.82)
Grippol plus, n (%)	9 (40.90)
Ultrix Quadri, n (%)	6 (27.27)

**Table 2:** Immunological efficacy of IIVs in hemodialysis patients (HAI assay).

Vaccine		UQ (n = 6)	GP (n = 9)	SG (n = 7)	CPMP Threshold Value *
Percentage of volunteers with AB titer ≥ 1:40, % (CI), Before vaccination (Day 0)	H1	0 (0, 0)	22 (0, 49)	43 (6, 80)	
	H3	17 (0, 46)	44 (12, 77)	29 (0, 62)	
	BV	17 (0, 46)	11 (0, 32)	14 (0, 40)	
	BY	0 (0, 0)	11 (0, 32)	0 (0, 0)	
Percentage of volunteers with AB titer ≥ 1:40, % (CI), Post-vaccination seroprotection rate (Day 21)	H1	100 (100, 100)	100 (100, 100)	71 (38, 105)	>60%
	H3	100 (100, 100)	100 (100, 100)	100 (100, 100)	
	BV	67 (29, 104)	78 (51, 105)	71 (38, 105)	
	BY	67 (29, 104)	44 (12, 77)	43 (6, 80)	
Fold increase in AB titers, Day 21 (CI)	H1	32 (9, 113)	16 (7.1, 36.2)	5.4 (1.7, 16.9)	>2
	H3	25.4 (14, 45)	10.1 (5.3, 19.1)	6.6 (1.9, 22.9)	
	BV	5 (1.2, 21.1)	12.7 (4.1, 39.4)	6.6 (2.7, 15.7)	
	BY	9 (2.9, 27.9)	22.2 (1.1, 4.3)	2.4 (1, 6.1)	
Percentage of volunteers with Seroconversion rate, % (CI)	H1	83 (54, 113)	89 (68, 100)	57 (20, 94)	>30%
	H3	100 (100, 100)	89 (68, 100)	71 (38, 100)	
	BV	67 (29, 100)	78 (51, 100)	71 (38, 100)	
	BY	83 (54, 100)	33 (3, 64)	43 (6, 80)	

* Threshold values for the population over 60 years of age. GP—Grippol plus, SG—Sovigripp, UQ—Ultrix Quadri. CPMP—European Committee on Patented Medicines (CPMP/BWP/214/1996). CI—confidential interval.

**Table 3:** Characteristics of the neutralizing antibody response to IIV vaccination in hemodialysis patients (MNA).

Vaccine		UQ (n = 6)	GP (n = 9)	SG (n = 7)	CPMP Threshold Value *
Percentage of volunteers with AB titer ≥ 1:40, % (CI), Before vaccination (Day 0)	H1	0 (0, 0)	11 (0, 32)	14 (0, 40)	
	H3	0 (0, 0)	0 (0, 0)	0 (0, 0)	
	BV	17 (0, 46)	11 (0, 32)	14 (0, 40)	
	BY	17 (0, 46)	22 (0, 49)	0 (0, 0)	
Percentage of volunteers with AB titer ≥ 1:40, % (CI), Post-vaccination seroprotection rate (Day 21)	H1	100 (100, 100)	89 (68, 100)	57 (20, 94)	>60%
	H3	83 (54, 100)	56 (23, 88)	14 (0, 40)	
	BV	50 (10, 90)	56 (23, 88)	57 (20, 94)	
	BY	83 (54, 100)	44 (12, 77)	43 (6, 80)	
Fold increase in AB titers, Day 21 (CI)	H1	22.6 (8, 63.9)	10.1 (4.9, 20.6)	3.6(1.9, 6.8)	>2
	H3	7.1 (3.2, 16.1)	7.4 (4.2, 13.2)	3 (1.4, 6.5)	
	BV	4.5 (1, 19.4)	5.4 (1.9, 15.8)	5.9 (1.8, 19.4)	
	BY	10.1 (4.4, 23.2)	1.7 (0.9, 3.2)	3 (1.2, 7.5)	
Percentage of volunteers with Seroconversion rate, % (CI)	H1	100 (100, 100)	89 (68, 109)	57 (20, 94)	>30%
	H3	83 (54, 100)	89 (68, 109)	57 (20, 94)	
	BV	50 (10, 90)	67 (36, 97)	71 (38, 100)	
	BY	83 (54, 100)	22 (0, 49)	43 (6, 80)	

* Threshold values for the population over 60 years of age. GP—Grippol plus, SG—Sovigripp, UQ—Ultrix Quadri. CPMP—European Committee on Patented Medicines (CPMP/BWP/214/1996). CI—confidence interval.

**Table 4:** Immunological efficacy of the IIVs in hemodialysis (HD) patients in comparison to groups of healthy volunteers (HAI).

Vaccine		HD (n = 22)	Healthy >60 (n = 42)	Healthy 18–60 (n = 34)
Percentage of volunteers with AB titer ≥ 1:40, % (CI), Before vaccination (Day 0)	H1	23 (5, 40)	38 (23, 53)	47 (30, 64)
	H3	32 (12, 51)	24 (11, 37)	29 (14, 45)
	BV	14 (0 ^#^, 28)	10 (1, 18)	21 (7, 34)
	BY	5 (0, 13)	5 (0, 11)	24 (9, 38)
Percentage of volunteers with AB titer ≥ 1:40, % (CI), Post-vaccination seroprotection rate (Day 21)	H1	91 (79, 103)	86 (75, 96)	94 (86, 102)
	H3	100 (100, 100)	83 (72, 95)	91 (82, 101)
	BV	73 (54, 91)	38 (23, 53)	68 (52, 83)
	BY	50 (29, 71)	31 (17, 45)	56 (39, 73)
Fold increase in AB titers, Day 28 (CI)	H1	13.7 (7.2, 26)	6.9 (4.2, 11.4)	11.5 (5.7, 23.2)
	H3	11.3 (6.7, 19.2)	5.6 (3.6, 8.9)	10.2 (5.4, 19.3)
	BV	8 (4.1, 15.4)	2.3 (1.8, 3)	3.3 (2.1, 5.1)
	BY	3.3 (1.9, 5.8)	2.4 (1.7, 3.2)	2.2 (1.4, 3.3)
Percentage of volunteers with Seroconversion rate, % (CI)	H1	77 (60, 95)	67 (52, 81)	74 (59, 88)
	H3	86(72, 101)	60 (45, 74)	76 (62, 91)
	BV	73 (54, 91)	36 (21, 50)	50 (33, 67)
	BY	50 (29, 71)	36 (21, 50)	32(17, 48)

^#^—negative values within the CI range were replaced by 0. CI—confidence interval. Light gray shading indicates data where the CIs do not overlap.

**Table 5:** Baseline characteristics of subjects (hemodialysis patients 2023–2024).

Demographic Characteristics	Total n = 71
Age, mean (min-max)	57.6 (25–85)
Female, n (%)	35 (49.3)
Male, n (%)	36 (50.7)
**Primary kidney disease**	
Chronic glomerulonephritis, n (%)	26 (36.62)
Polycystic kidney disease, n (%)	13 (18.31)
Chronic tubulointerstitial nephritis, n (%)	11 (15.49)
Hypertension, n (%)	4 (5.63)
Type 1 diabetes, n (%)	3 (4.23)
Type 2 diabetes, n (%)	3 (4.23)
Gouty nephritis, n (%)	3 (4.23)
Alport syndrome, n (%)	3 (4.23)
Connective tissue disease, n (%)	3 (4.23)
Abnormality of kidney development, n (%)	1 (1.41)
Kidney Cr, Nephrectomy, n (%)	1 (1.41)
spKt/V, mean (95%CI)	1.55 (1.49–1.60)
Time on dialysis, years, mean (min-max)	6.32 (0.06–29.28)
**Laboratory parameters**	
Hemoglobin, g/L, mean (95%CI)	112.20 (109.50–114.90)
Albumine, g/L, mean (95%CI)	40.62 (39.89–41.35)
P, mmol/L, mean (95%CI)	1.81 (1.67–1.96)
Ca, mmol/L (95%CI)	2.24 (2.19–2.29)
**Vaccination**	
First year vaccination, n (%)	28 (39.44)
Second year vaccination, n (%)	34 (47.89)
Third year vaccination, n (%)	9 (12.67)
Flu-M Tetra, n (%)	71 (100)

**Table 6:** Immunogenic efficacy of vaccination in hemodialysis, depending on the previous vaccination status (season 2023–2024, HAI data).

Vaccine		All (n = 58) *	1Y (n = 22)	2Y (n = 29)	CPMP Threshold Value *
Percentage of volunteers with AB titer ≥ 1:40, % (CI), Before vaccination (Day 0)	H1	33 (21, 45)	18 (2, 34)	38 (20, 56)	
	H3	40 (27, 52)	32 (12, 51)	41 (23, 59)	
	BV	26 (15, 37)	5 (0, 13)	41 (23, 59)	
	BY	40 (27, 52)	23 (5, 40)	55 (37, 73)	
Percentage of volunteers with AB titer ≥ 1:40, % (CI), Post-vaccination seroprotection rate (Day 28)	H1	91 (84, 99)	91 (79, 100)	93 (84, 100)	>60%
	H3	83 (73, 92)	86 (72, 100)	79 (65, 94)	
	BV	71 (59, 82)	55 (34, 75)	86 (74, 99)	
	BY	86 (77, 95)	91 (79, 100)	90 (79, 100)	
Fold increase in AB titers, Day 28 (CI)	H1	7.2 (5.1, 10)	13.2 (7.9, 22)	5.7 (3.5, 9.2)	>2
	H3	5 (3.7, 6.8)	8.8 (5.3, 15)	3.9 (2.6, 5.9)	
	BV	4.8 (3.5, 6.6)	5.8 (3.2, 11)	4.5 (2.9, 6.9)	
	BY	5.1 (3.6, 7.3)	11.7 (6.3, 22)	3.4 (2.3, 5)	
Percentage of volunteers with Seroconversion rate, % (CI)	H1	72 (61, 84)	86 (72, 100)	72 (56, 89)	>30%
	H3	67 (55, 79)	86 (72, 100)	62 (44, 80)	
	BV	62 (50, 75)	68 (49, 88)	62 (44, 80)	
	BY	59 (46, 71)	86 (72, 100)	41 (23, 59)	

*—The analysis was performed only for volunteers with complete datasets. 1Y—group of hemodialysis patients vaccinated in the 2023/2024 season; 2Y—group of hemodialysis patients vaccinated in both the 2022/2023 and 2023/2024 seasons. CPMP—European Committee on Patented Medicines (CPMP/BWP/214/1996). CI—confidence interval. Light gray shading indicates data where the CIs do not overlap.

**Table 7:** Immunogenic efficacy of vaccination in dialysis patients depending on the previous vaccination status (season 2023–2024, MNA data).

Vaccine		All (n = 58) *	1Y (n = 22)	2Y (n = 29)	CPMP Threshold Value *
Percentage of volunteers with AB titer ≥ 1:40, % (CI), Before vaccination (Day 0)	H1	53 (41, 66)	27 (9, 46)	69 (52, 86)	
	H3	9 (1, 16)	0 (0, 0)	17 (3, 31)	
	BV	24 (13, 35)	5 (0, 13)	41 (23, 59)	
	BY	19 (9, 29)	14 (0, 28)	24 (9, 40)	
Percentage of volunteers with AB titer ≥ 1:40, % (CI), Post-vaccination seroprotection rate (Day 28)	H1	97 (92, 100)	91 (79, 100)	100 (100, 100)	>60%
	H3	57 (44, 70)	59 (39, 80)	66 (48, 83)	
	BV	81 (71, 91)	68 (49, 88)	90 (79, 100)	
	BY	76 (65, 87)	82 (66, 98)	76 (60, 91)	
Fold increase in AB titers, Day 28 (CI)	H1	6.9 (4.9, 9.8)	15.5 (8.5, 28)	4.6 (3.1, 6.9)	>2
	H3	4.6 (3.5, 6.1)	6.2 (3.5, 11)	4 (2.8, 5.7)	
	BV	6.3 (4.4, 9)	7.5 (3.9, 14)	6 (3.7, 9.8)	
	BY	5.1 (3.9, 6.8)	8.8 (5.8, 13)	4.3 (2.9, 6.3)	
Percentage of volunteers with Seroconversion rate, % (CI)	H1	74 (63, 85)	91 (79, 100)	66 (48, 83)	>30%
	H3	64 (51, 76)	64 (44, 84)	66 (48, 83)	
	BV	69 (57, 81)	73 (54, 91)	69 (52, 86)	
	BY	66 (53, 78)	86 (72, 100)	59 (41, 77)	

*—The analysis was performed only for volunteers with complete datasets. 1Y—group of hemodialysis patients vaccinated in the 2023/2024 season; 2Y—group of hemodialysis patients vaccinated in both the 2022/2023 and 2023/2024 seasons. CPMP—European Committee on Patented Medicines (CPMP/BWP/214/1996). CI—confidence interval. Light gray shading indicates data where the CIs do not overlap.

## Data Availability

All data supporting reported results are available upon request.

## Supplementary Materials

The following supporting information can be downloaded at https://www.mdpi.com/article/10.3390/vaccines14010063/s1: Figure S1: Polyfunctional memory T-cell gaiting strategy; Figure S2: Tfh gating strategy; Figure S3: B-cell gaiting strategy.
