# Supplementary material for: Influenza Vaccine Immunogenicity in Hemodialysis Patients
Source: Vaccines (Basel). 2026 Jan 4;14(1):63. doi: 10.3390/vaccines14010063 (PMC12846267; doi:10.3390/vaccines14010063)
Supplement: Supplementary file 1 [file vaccines-14-00063-s001.zip › vaccines-4048816-supplementary.pdf]

vaccines

MDPI

Supplementary Materials

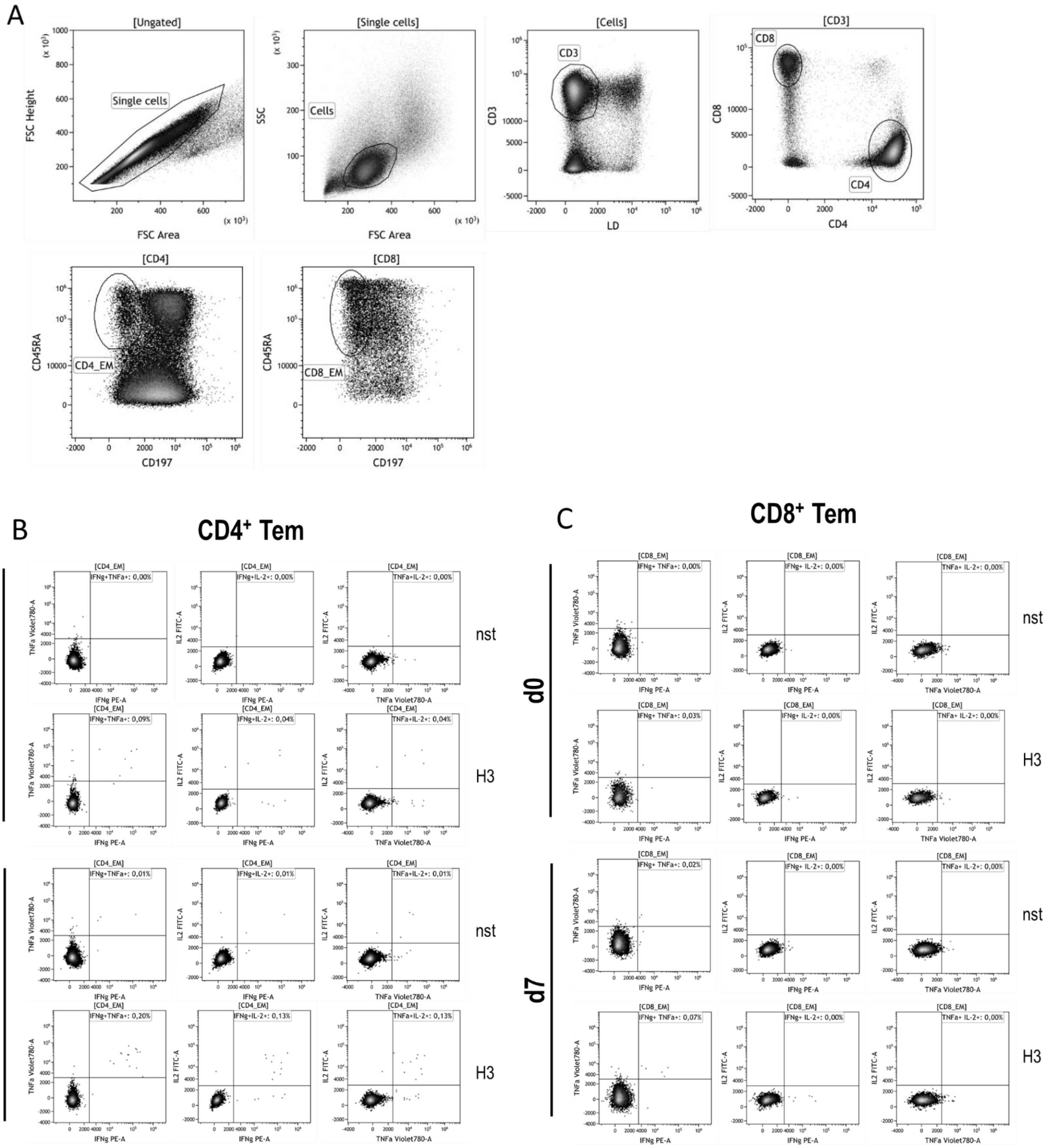

Figure S1. Polyfunctional memory T-cell gaiting strategy. Based on the characteristics of forward and side light scattering (FSC/SSC) and Zombie Aqua fluorescence, non-viable cells were excluded from the analysis. The population of live cells was divided into two main T-lymphocyte subpopulations: T-helper cells (CD4+) and cytotoxic T-cells (CD8+). Based on the presence of CD45RA and CD197 markers, subpopulations of central (CM) and effector (EM) memory T-cells were identified (**A**). The population of CD4+ and CD8+ effector memory T cells was further characterized for their ability to produce cytokines (IFNγ, TNFα, IL2) intracellularly. Representative plots of CD4+Tem cells producing cytokines (**B**). Representative plots of CD4+Tem cells producing cytokines (**C**).

vaccines

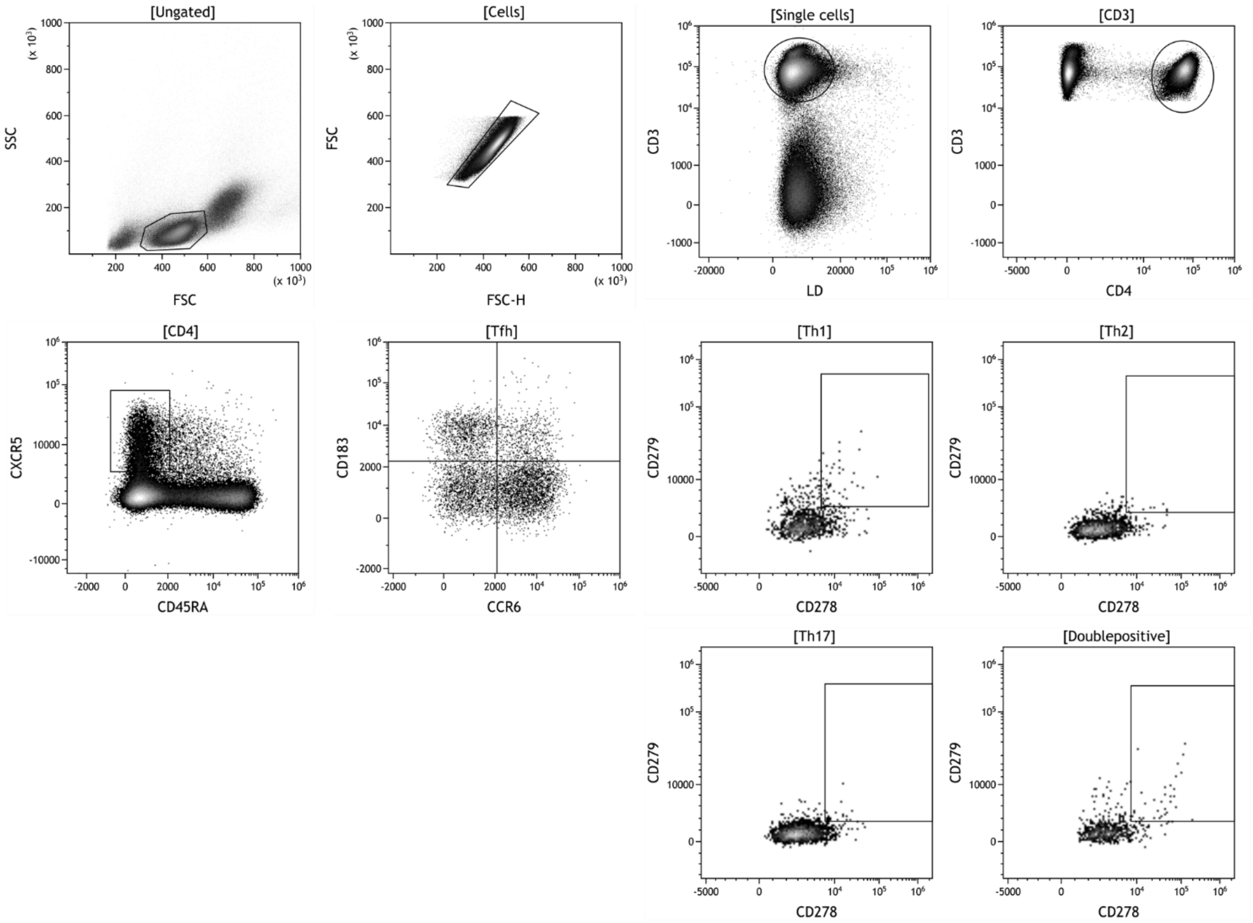

Figure S2. Tfh gating strategy. Non-viable cells were discriminated based on forward and side scattered light (FSC/SSC) parameters, doublets exclusion (FSC-H/FSC-A), and the degree of binding of the viability marker Zombie Aqua. Tfh cells were identified from the parent population of live CD4+ T lymphocytes as CD45RA$^-$ cells expressing CXCR5. Subsequently, the total Tfh population was subdivided based on the differential expression of CCR6 (CD196) and CXCR3 (CD183) markers into Tfh1 (CXCR3$^+$ CCR6$^-$), Tfh2 (CXCR3$^-$ CCR6$^-$), and Tfh17 (CXCR3$^-$ CCR6$^+$) subpopulations. The activation of Tfh within these subpopulations was assessed based on the simultaneous expression of ICOS (CD278) and PD-1(CD279).

vaccines

MDPI

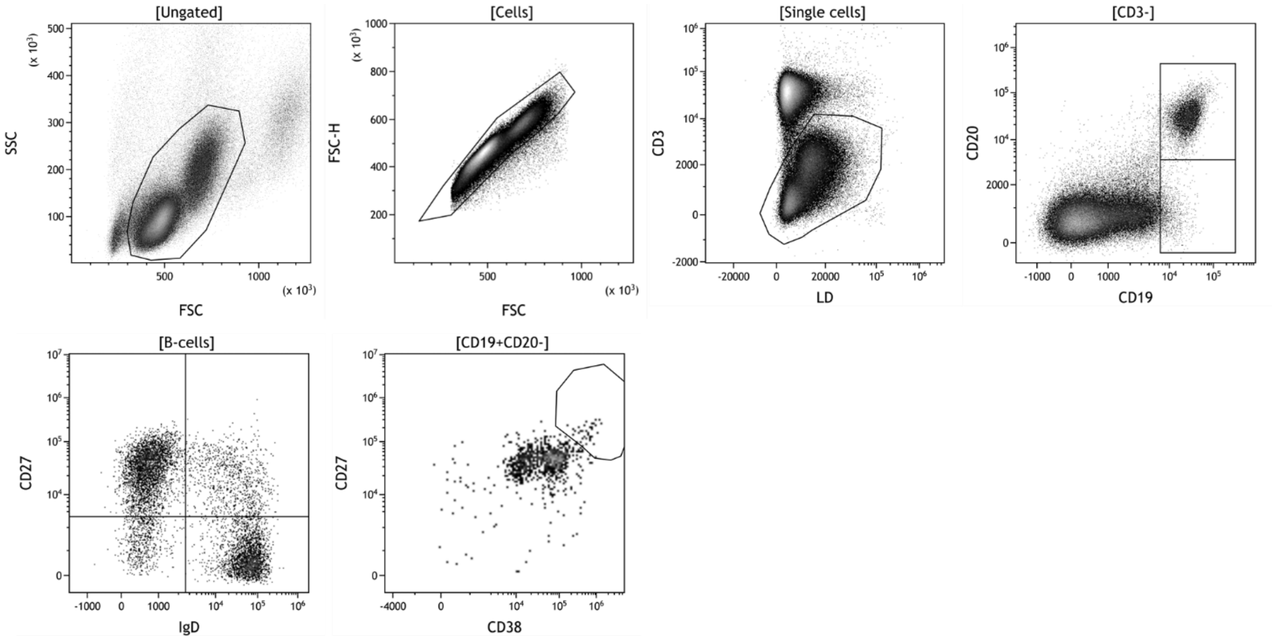

Figure S3. B-cell gaiting strategy. Non-viable cells and CD3+ lymphocytes were excluded from the analysis based on characteristics of forward and side scatter (FSC/SSC), exclusion of doublets (FSC-H/FSC-A), Zombie Aqua fluorescence, and the presence of CD3. Within the B-lymphocyte population ($CD3^{-}$ $CD19^{+}CD20^{+}$), the following subsets were identified: naive B cells (CD27-IgD+), unswitched memory B cells (CD27+IgD+), switched memory B cells (CD27+IgD-), and effector memory B cells (CD27-IgD-). Plasmablasts were identified as (CD20-$CD38^{hi}CD27^{hi}$).
